# Rapid Assessment of Lipidomics Sample Purity and Quantity Using Fourier-Transform Infrared Spectroscopy

**DOI:** 10.3390/biom12091265

**Published:** 2022-09-08

**Authors:** Harley Robinson, Jeffrey Molendijk, Alok K. Shah, Tony Rahman, Gregory J. Anderson, Michelle M. Hill

**Affiliations:** 1 QIMR Berghofer Medical Research Institute, Herston, Brisbane, QLD 4006, Australia; 2 Department of Gastroenterology & Hepatology, The Prince Charles Hospital, Rode Road, Brisbane, QLD 4032, Australia; 3 Centre for Clinical Research, Faculty of Medicine, The University of Queensland, Herston, Brisbane, QLD 4006, Australia

**Keywords:** lipids, phospholipids, sphingolipids, triglycerides, FTIR, mass spectrometry, chemical contaminants

## Abstract

Despite the increasing popularity of liquid chromatography–mass spectrometry (LC-MS)-based lipidomics, there is a lack of accepted and validated methods for lipid extract quality and quantity assessment prior to LC-MS. Fourier-Transform Infrared Spectroscopy (FTIR) has been reported for quantification of pure lipids. However, the impact of complex lipid sample complexity and purity on total lipid quantification accuracy has not been investigated. Here, we report comprehensive assessment of the sample matrix on the accuracy of lipid quantification using Attenuated Total Reflectance (ATR)-FTIR and establish a simple workflow for lipidomics sample quantification. We show that both pure and complex lipids show characteristic FTIR vibrations of CH- and C=O-stretching vibrations, with a quantitative range of 40–3000 ng and a limit of detection of 12 ng, but sample extraction method and local baseline subtraction during FTIR spectral processing significantly impact lipid quantification via CH stretching. To facilitate sample quality screening, we developed the Lipid Quality (LiQ) score from a spectral library of common contaminants, using a ratio of peak heights between CH stretching vibrations maxima and the collective vibrations from amide/amine, CH-stretching minima and sugar moieties. Taking all tested parameters together, we propose a rapid FTIR workflow for routine lipidomics sample quality and quantity assessment and tested this workflow by comparing to the total LC-MS intensity of targeted lipidomics of 107 human plasma lipid extracts. Exclusion of poor-quality samples based on LiQ score improved the correlation between FTIR and LC-MS quantification. The uncertainty of absolute quantification by FTIR was estimated using a 795 ng SPLASH LipidoMix standard to be <10%. With low sample requirement, we anticipate this simple and rapid method will enhance lipidomics workflow by enabling accurate total lipid quantification and normalization of lipid quantity for MS analysis.

## 1. Introduction

Biological lipids are a heterogeneous group of non-polar compounds that perform a range of critical functions from compartmentalization to energy storage to signaling [1]. Unlike genes and proteins, biological lipid synthesis is not template driven; the dynamic lipidome is an interaction between lipid metabolic enzyme activity and substrate accessibility which is influenced by the environment (including diet). Biological lipids are classified by their chemical structure into eight classes based on the hydrophobic and hydrophilic components of the lipid [2]. However, the structure–function relationship of lipids goes beyond the class, with features such as fatty acid chain length, number and position of saturation (double bond) influencing lipid function [3]. Furthermore, the relative composition of lipid classes modifies the physical properties of membranes [3]. Therefore, the goals of lipidomics are to profile the composition of the lipidome and to detect and quantify lipid species. While advances in mass spectrometry technology and development of analytical and lipid-specific informatics methods have enabled high-throughput lipidomics, a validated method of sample quality control is yet to be established.

In principle, the steps in the workflow for lipidomics should parallel the other, more developed omics, and comprise sample extraction, quality control, and total lipid quantification steps prior to normalization of lipid sample amount for molecular profiling. However, sample quality control and normalization of loading by the total lipid amount in a sample is currently absent from the recommendations for good practice in lipidomics [4], possibly due to the lack of validated, fit-for-purpose, sample-preserving methods for lipid sample quality control prior to mass spectrometry analysis. Without total lipid quantification, sample volume (e.g., serum/plasma) or weight (e.g., tissue) have been used as the basis of normalization. While this approach is appropriate for studies using well-characterized sample types in cross-sectional or well-controlled studies, a sample quality control and quantification step is essential for novel, less-controlled sample types; for example, materials derived from foodstuffs, plant samples and wildlife. 

Despite the heterogeneity of biological lipids, we reasoned that total lipid quantity and quality assessment may be accomplished through spectroscopic measurements of common lipid structures such as hydrocarbons and ester bonds, which are generally absent in contaminating biochemicals. A similar application is used in the RNA sequencing workflow, where preparations of RNA from any source are routinely assessed for quality and quantity via UV/VIS spectroscopy (NanoDrop spectrometers) using a generalized metric of RNA-specific peaks (absorbance at 260 nm, A260) despite different absorbance peaks for adenosine, uridine (260 nm), guanosine (254 nm), and cytosine (271 nm) [5]. Ratios between A260 and absorbance of known interfering molecules (A230, A280) are used to assess sample purity [6]. 

For lipid quantification, there is an existing commercial product (DirectDetect^TM^) [7] which uses Fourier-transform infrared (FTIR) spectroscopy with an external standard to quantify lipids based on CH symmetric stretching vibration between 2870 and 2840 cm^−1^ [8]. While this simple formula is accurate for pure lipids, the quantification accuracy of complex lipid samples/mixtures, and the potential impact of different lipid extraction methods and biochemical contamination on total lipid quantification by FTIR have not yet been fully assessed. Since the presence of other (bio)chemicals in the sample can influence IR absorbance for CH stretching due to the masking effect [9], and lipidomics experiments almost always analyze complex (rather than pure) lipids, a technical assessment and validation of complex lipid quantification by FTIR are essential prior to adoption into the workflow. 

Here, we conducted a systematic evaluation of the parameters that impact total complex lipid mixture quantification by FTIR and developed new formulae to facilitate the use of FTIR for lipid sample quality assessment. We chose the Attenuate Total Reflectance (ATR) sampling method for FTIR because it requires just one microliter of MS-ready sample without additional sample preparation. The organic solvent from the lipid extracts readily evaporates on the ATR sensor, allowing rapid FTIR data acquisition. Firstly, we examined the impact of sample extraction method and FTIR spectra local baseline correction on the ensuing lipid quantification against the external lipid standard curve using either a single lipid or a lipid mixture. For this assessment, we compared FTIR spectra of a cellular lipid extract using commonly used monophasic and biphasic extraction methods to pure lipid standard and the mixed cell extract (with all biomolecules). Secondly, we assessed common biochemical contaminants that may occur during sample preparation and lipid extraction (e.g., incomplete metabolite removal, trace sucrose, detergents), or due to contaminants in the solvent or tubes (e.g., PEG). We titrated a range of common biochemical contaminants to a pure lipid extract to assess the sensitivity ATR-FTIR for contaminant detection. Based on the results of these extensive investigations, we developed a lipid quality (LiQ) score to detect contamination of lipid samples. Finally, the LiQ score was evaluated on a set of plasma samples with comparison to LC-MS-based lipidomics. 

## 2. Materials and Methods

**Figure 1:**
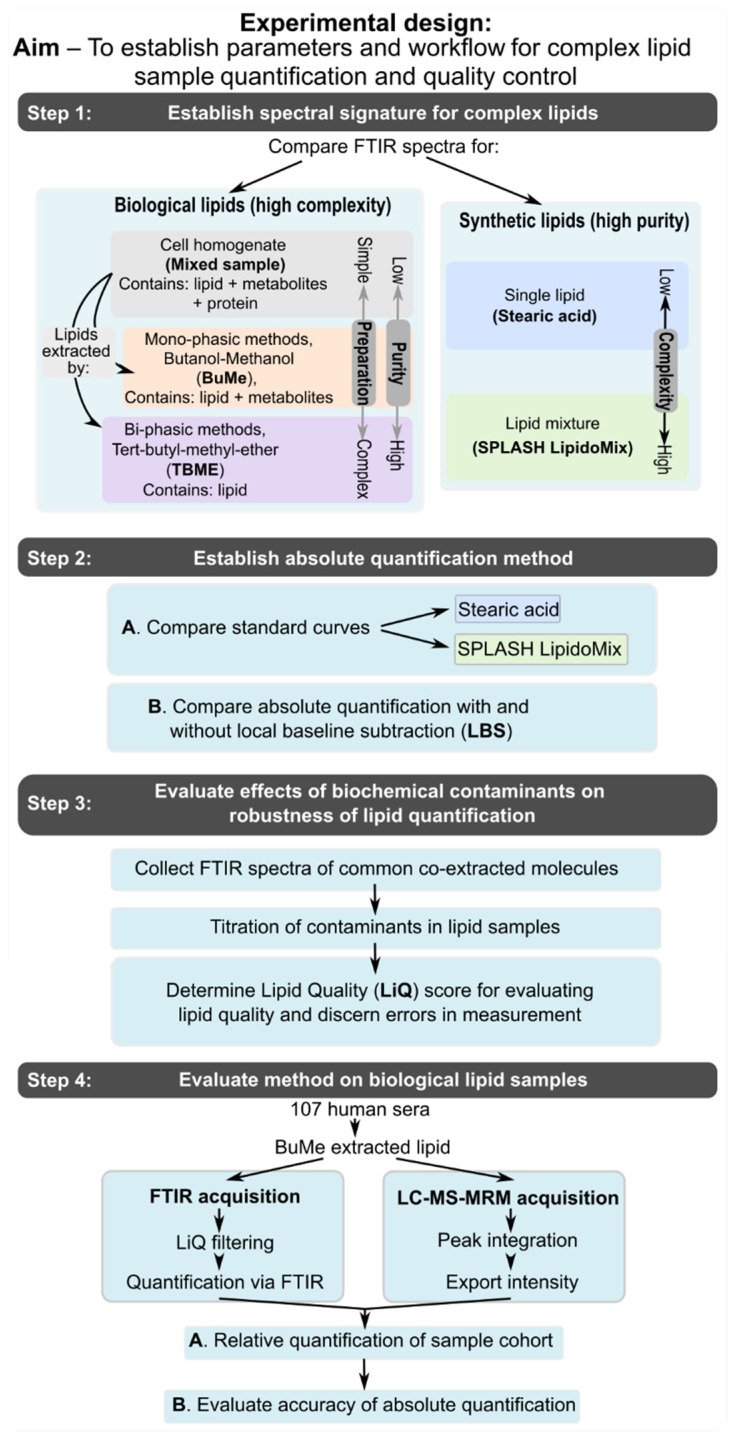
Experimental design evaluating FTIR spectroscopy parameters for complex lipid mixture quantification and quality control.

Figure 1 depicts the experimental design formulated to assess the parameters for using ATR-FTIR spectroscopy for quality and quantity assessment of complex lipid mixtures, which are the starting materials for lipidomics. 

### 2.1. Materials

Stearic acid (S4751), sucrose (84097), glucose (G8270), galactose (G0750), NP-40 (542334), Triton-X100 (T8787), sodium deoxycholate (D6750), 1-butanol (34867), 2,6-di-tert-butyl-4-methyl-phenol (BHT, B1378), tert-butyl-methyl-ether (TBME, 34875), 2-aminoanthracene (A38800) and Val-Tyr-Val (V8376) were purchased from Sigma Aldrich (Castle Hill, Australia). RNA primer (Qiagen, MS00003556) was purchased from Qiagen (Clayton, Australia). SPLASH LipidoMix (330707) was purchased from Avanti Polar Lipids, inc. (Alabaster, AL, USA). Agilent API-TOF Reference Mix (G1969-85001), 250 µL PP inserts with graduation (#5190-4073), and PTFE/silicone rubber septa (#5182-0731) were ordered from Agilent Technologies (Mulgrave, VIC, Australia). Methanol (A456-4), 2-propanol (A451-4), and acetonitrile (A955-4) were purchased from Thermo Fisher Scientific (Scoresby, Australia). The Synergy UV Water Purification System was used to filter MilliQ water (Merck Millipore, Burlington, MA, USA).

### 2.2. Cell Culture and Lipid Extract Preparation

PC3 (RRID: CVCL_0035) cells were grown in 5% FBS (Bovogen, SFBS-FR)/RPMI-1460 (Gibco, 11875119) culture medium at 37 °C, 5% CO_2_. Cells were transferred into 10 cm Petri dishes, harvested by cell scraping, and counted using a hemocytometer. To prepare three extracts with varying levels of contamination, aliquots of one million cells were prepared, pelleted, and washed with PBS, and then processed using one of the following methods. For the “Mixed sample” of all cellular components, the cell pellet was resuspended with 300 µL butanol:methanol, followed by immediate analysis of the cell extract by FTIR spectroscopy. In case of the “BuMe” extract, the cell pellet was resuspended with 300 µL butanol:methanol, then incubated overnight at −20 °C. Protein was then removed by centrifugation at 16,000× *g* for 30 min at 4 °C, and the supernatant was collected in a separate tube and analyzed using FTIR spectroscopy. For the “TBME” extract, the cell pellet was resuspended in 200 µL of chilled methanol, sonicated for 1 min, and incubated overnight at −20 °C. TBME (tert-butyl-methyl-ether, 700 µL) was added and the sample was vortexed before adding 180 µL of milliQ water. After mixing, the sample was centrifuged at 16,000× *g* for 30 min at 4 °C and the upper (lipid extract) and middle (metabolite extract, for quality control analysis) phases were collected into individual tubes. The lipid extract was then dried, resuspended in 300 µL butanol:methanol, and analyzed via FTIR spectroscopy. The remaining protein pellet was washed and resuspended (50 µL) in PBS, and the amount of protein was quantified by BCA assay for use in quality control analyses. 

### 2.3. Preparation of Contaminant Samples

Stock mixtures of each contaminant were generated via the following methods. Solid sucrose, glucose, and galactose were resuspended in 10 µL of MilliQ water before dilution in 3:1 butanol:methanol to a final volume of 1 mL and concentration of 1 µg/µL. Protein extracted from cell lysates was diluted in 3:1 butanol:methanol to provide a 1 mL stock solution with a protein concentration of 1 µg/µL. Due to the precipitation of protein and sugars in organic solvents, these samples were thoroughly vortexed before each use, measurement, or dilution. Solid detergent (sodium deoxycholate) were resuspended in 3:1 butanol:methanol to a concentration of 1% (*w*/*v*), whereas liquid detergent (Triton X-100, NP-40) was diluted to 1% (*v*/*v*) in 3:1 butanol:methanol. Lyophilised RNA primer was resuspended in 10 µL MilliQ water and diluted to 1 µg/µL (200 µL) in 3:1 butanol:methanol, and its concentration confirmed using a NanoDrop 2000/2000c spectrometer (Thermo Fisher Scientific). Metabolites extracted from 10^7^ cells (method described above) were collected from the aqueous phase of a TBME extraction (380 µL), dried, and resuspended in 380 µL 3:1 butanol:methanol. Lipids purified by TBME extraction were then quantified by ATR-FTIR spectroscopy and diluted to 1 µg/µL for use as pure lipid diluent for quality-control analysis. Lipids extracted using the BuMe method were diluted using the same dilution factor as TBME lipids. 

Dilutions for quality control analysis were prepared by spiking variable amounts of contaminant stock into 10 µL pure lipid extract, followed by drying and resuspension in 10 µL 3:1 butanol:methanol. This ensures a constant lipid concentration of 1 µg/µL without dilution from the contaminant that has been spiked in. 

### 2.4. Human Plasma Sample Collection

Deidentified plasma samples from a cohort of patients with chronic liver disease were used for proof-of-concept application of lipid extract quality assessment. These samples were collected following informed consent from the participants. The work was approved by the research ethics committees of the Prince Charles Hospital (HREC/15/QPCH/202) and QIMR Berghofer Medical Research Institute (P2352), and abided by the Declaration of Helsinki principles. Blood was collected using EDTA as the anticoagulant. Following centrifugation of the samples, plasma was collected and stored at −80 °C.

### 2.5. Human Plasma Sample Preparation

Plasma samples were thawed on ice prior to metabolite and lipid extractions. All pipetting steps were performed in a cold room or on ice. Plasma (30 µL) was mixed with 270 µL of butanol/methanol (1:1 *v*/*v*) containing 10 mM ammonium formate, 50 µg/mL BHT, and 1.5 µL SPLASH internal standard mixture. Samples were agitated using a thermomixer and sonicated (25 °C, 850 rpm, 1 h). Samples were centrifuged at 16,000× *g* (20 °C, 15 min) before aliquoting 100 µL of the supernatant for mass spectrometry analysis. For FTIR spectra acquisition, 100 µL of butanol:methanol lipid extracts were aliquoted into a Greiner V-bottom 96-well plate and dried using an evaporative sample concentrator (Genevac EZ-2, Marshall scientific). The samples were reconstituted in 12 µL of butanol:methanol (1:1). 

### 2.6. ATR-FTIR Spectroscopy

An Agilent Cary 630 fitted with an ATR module was used for the acquisition of spectra. Mid-infrared spectra (4000–650 cm^−1^) were collected at a resolution of 8 cm^−1^ using 64 scans per acquisition. The detector stage was cleaned with 80% ethanol, and the background spectra (ambient room air at 21 °C) were collected. A lipid sample (1 μL) or standard in butanol:methanol mixture was applied to the detector and allowed to air dry (~30 s). Lipid standards were prepared by serial dilution of stearic acid and Avanti SPLASH LipidoMix in butanol:methanol. All spectra were baseline corrected using the baseline algorithm built into the Agilent MicroLab Expert software with set regions 2031–1865 cm^−1^ and 3971–3799 cm^−1^. Note that the baseline correction adjusts the entire spectra baseline to the experimental baseline (blank), which is different to the local baseline subtraction procedure that we test during this study. Local baseline subtraction was not applied to all spectra. The spectra were then exported from MicroLab Expert (Agilent) software as CSV files. 

### 2.7. LC-MS Lipidomics

Targeted lipidomics experiments were performed following previously published methodologies [10]. Briefly, an Agilent Technologies 1290 Infinity II UHPLC system with an Agilent ZORBAX eclipse plus C18 column (2.1 × 100 mm 1.8 µm) (#959758-902) and guard column (#821725-901), coupled online with an Agilent 6470 triple quadrupole system, was used for the targeted lipidomics experiments. The instrument was tuned in positive ionization mode and unit resolution. Buffer A contained 10 mM ammonium formate in water/acetonitrile/isopropanol (50:30:20% *v*/*v*/*v*), whereas buffer B contained 10 mM ammonium formate in water/acetonitrile/isopropanol (1:9:90% *v*/*v*/*v*). A multi-wash procedure was performed prior to each sample injection. In this procedure, the needle was washed and needle seat back flushed with isopropanol, MilliQ water, and acetonitrile to reduce sample carryover. 

The source nitrogen gas temperature was set to 175 °C at a flow of 11 L/min and a sheath gas temperature of 250 °C at a flow of 10 L/min. The capillary voltage was set to 3500 V and nozzle voltage to 0 V for positive mode, and the nebulizer operated at 20 psi. Check tunes were performed in wide, unit, and enhanced modes prior to each experiment to confirm the performance of the mass spectrometer. The quadrupole was tuned to reference masses 118.09, 322.05, 622.03, 922.01, and 1221.99 in positive ionization mode; 112.99, 302.00, 601.98, 1033.99, and 1333.97 in negative ionization mode. 

The instrument was operated in dynamic MRM mode using the transitions published by Huynh et al. [10], including LipidoMix internal standards. Six microliters of sample were injected per acquisition. Acquired data were imported into Skyline (MacCoss Lab, Department of Genome Sciences, University of Washington, Seattle, DC, USA) [11]. Peak integration was automated, but it was manually confirmed and corrected if required. Internal standard retention time was used to confirm correct peak integration of lipids belonging to the same class. Peak areas were exported from Skyline for further analysis in R (R Foundation for Statistical Computing, Vienna, Austria) [12]. Total intensity (TI) values were generated by summing all lipid species intensities per acquisition and adjusted to reflect differences in concentration between MS measurements (6 µL of 100 µL plasma lipid sample, 6% total sample measured per acquisition) and FTIR spectroscopy (1 µL of concentrated plasma lipid sample, 8.33% total sample measured per acquisition). 

### 2.8. Data Analysis and Statistics

**Figure 2:**
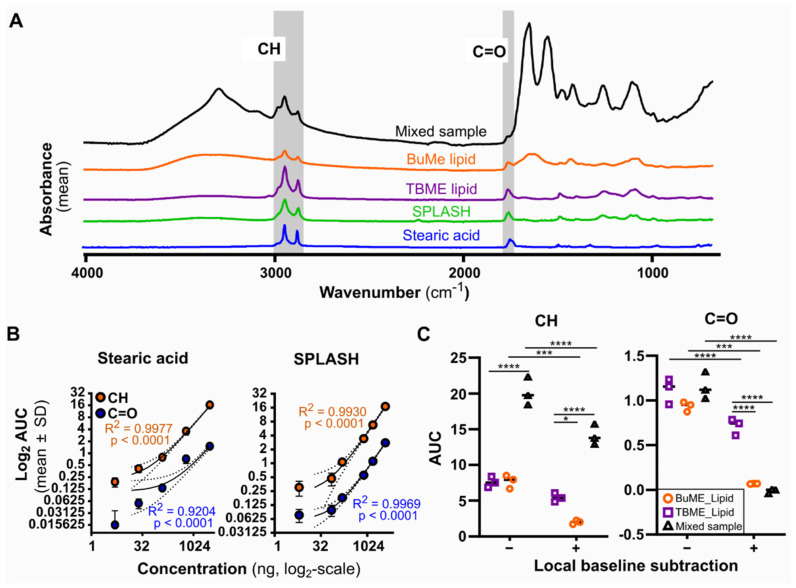
Assessment of lipid quantification by ATR-FTIR spectroscopy—impact of different extraction methods, standards, and spectral processing. (**A**) Analysis of spectral features in extracted (BuMe, TBME) or commercial lipid (SPLASH, stearic acid) samples. Hydrocarbon (3000–2800 cm^−1^) and ester (1760–1710 cm^−1^) regions are highlighted in gray. (**B**) Titration of lipid standard by simple fatty acid (stearic acid) or complex lipid mixtures (SPLASH), expressed as a log–log graph. Linear regression analysis was performed between the area under the curve (log_2_AUC) of the CH and C=O regions and known lipid quantities (log_2_). *n* = 3. Linear regression was fitted, displaying 95% confidence interval. (**C**) Area under the curve (AUC) measurements of the CH and C=O regions either with (+) or without (−) local baseline subtraction; see Methods Section 2.8 for formula. Two-sided two-way ANOVA compared between measurements, where * *p* < 0.05, *** <0.001, **** <0.0001.

Method development and optimization analysis were completed using R (version 4.0.2) and Graphpad Prism software, where the final application utilized MicroLab Quant and Expert software. Baselined spectra were analyzed using R studio and the DescTools package (0.99.44). All analyses were completed on baselined spectra to standardize all spectra to the same baseline. No normalization was completed on these spectra due to the quantitative nature of the analysis. The area under the curve (AUC) was calculated for each desired region using the trapezoid method via the AUC function. For analysis in Figure 2C only, a local baseline was calculated using the minimum and maximum range values in the trapezoid area formula [area = (Height_Min_−Height_Max_)/2 × (Wavenumber_Max_−Wavenumber_Min_)] and subtracted from each AUC to result in a locally baselined AUC value. Mean baselined AUC and standard deviation were calculated across the technical replicates (*n* = 3−6) for each sample. Simple linear regression analysis and graphs were generated by Graphpad Prism (v8.4.3., GraphPad Software Inc., San Diego, CA, USA) for each standard lipid calibration curve. Signal to noise ratios (S/N), limits of detection (LOD) and quantitation (LOQ) were calculated using the International Conference on Harmonization method [13]. FTIR and mass spectrometry measures were compared by Pearson correlation (R value) and simple linear regression analysis (R^2^ and *p* values). Two-sided two-way ANOVA test comparing analysis parameters was conducted using GraphPad Prism, with Sidak’s multiple comparisons. Graph collation and diagrams were generated by Inkscape.

## 3. Results

### 3.1. Impact of Lipid Sample Purity on ATR-FTIR Spectra—Comparison of Lipid Extraction Methods

First, to establish the FTIR spectral signature for complex lipid mixtures, we compared the spectra of a single lipid (Stearic acid), a synthesized lipid mixture imitating human plasma composition (SPLASH LipidoMix), to extracts from a cell pellet with varying level of lipid purity. We chose two commonly used lipid extraction methods: monophasic butanol:methanol (BuMe) extraction, and biphasic tert-butyl methyl ether (TBME) extraction. In addition, the full cell extract (BuMe suspension) comprising all biomolecules was also analyzed (termed Mixed sample in Figure 2). Aliquots of one million cells from the same batch were used for each extraction method, as described in Methods Section 2.2. As shown in Figure 2A, the TBME extract and SPLASH LipidoMix had very similar spectra, indicating that TBME generates a highly pure lipid extract. The hydrocarbon (CH-stretching region in the 3000–2800 cm^−1^) and ester (C=O, 1760–1730 cm^−^^1^) peaks were detected, with no observable neighboring peaks that could confound area under the curve (AUC)-based quantification (Figure 2A). A titration of the SPLASH LipidoMix mixture showed linearity for both the CH and C=O regions, comparable to a simple saturated lipid stearic acid (an 18-chain hydrocarbon and a terminal carboxyl group), indicating a linear relationship between these regions and lipid abundance (Figure 2B). We further characterized the detection limits and signal-to-noise ratios (SNR) for absolute quantification using the CH and C=O peaks for each lipid calibration curve (Table 1). Good signal-to-noise ratios were observed, ranging from 2.525 (CH, steric acid) to 4.432 (C=O, steric acid). SPLASH LipidoMix had a consistent SNR of 3.071–3.333 for C=O and CH, respectively (Table 1). With the exception of C=O for stearic acid, excellent linearity was obtained in the measured range, with R^2^ > 0.96 (Figure 2B). The CH band had the better sensitivity, with very similar LOD (~12 ng) and LOQ with stearic acid and SPLASH LipidoMix calibration curves (~40 ng, Table 1). 

While these calibration curves demonstrate the quantification of pure lipid samples by FTIR, two of the complex lipid samples (Mixed sample and BuMe extract) present confounding neighboring peaks in both the CH and the C=O regions. Although TBME provided the purest lipid extract, the biphasic extraction method is not easily adapted to high-throughput procedures and is prone to accidental contamination during the procedure. In contrast, BuMe extraction is liquid-handler-friendly and has been widely used in lipidomics. Therefore, we next compared the relative quantification accuracy for either CH or C=O regions of the different extraction methods, using TBME extraction as benchmark. BuMe, TBME, and Mixed samples were expected to have the same total lipid quantity, and the same CH/C=O AUC, as lipids, were extracted from the aliquots of the same batch of cells. As shown in Figure 2C (left—local baseline subtraction), the unadjusted AUC of the CH region provided consistent relative quantification between TBME and BuMe extraction, but gave an overestimation in the Mixed sample due to the large contribution of the neighboring broad peak (3500–2500 cm^−1^). Surprisingly, the unadjusted C=O peak yielded similar AUCs across all three samples, despite the contribution of neighboring peaks in the BuMe and Mixed samples (Figure 2C, right—local baseline subtraction). However, the AUC for the C=O peak was drastically lower than the CH region. 

Finally, we evaluated local baseline subtraction, as it is often used as a standard data-processing step in FTIR-based quantification [7,14]. We compared the AUC values for both CH and C=O regions, with and without local baseline subtraction, calculated using the trapezoid area formula detailed in Methods Section 2.8. Comparing the AUC values between + and − local baseline subtraction in Figure 2C for each of the extraction methods, we observed significant differences for BuME and Mixed sample measurements for CH and C=O regions, with TBME-extracted lipid only differing in the C=O region (all *p* < 0.001). These results indicate previously unrecognized quantification errors for some samples when local baseline subtraction is automatically applied in FTIR-based lipid quantification. Taken together, our results caution against using local baseline subtraction, and suggest the potential use of the C=O peak for quantification of non-pure lipid extracts. 

### 3.2. ATR-FTIR Spectroscopy Differentiates Lipid from other Biological Materials for Quality-Control Assessment

**Figure 3:**
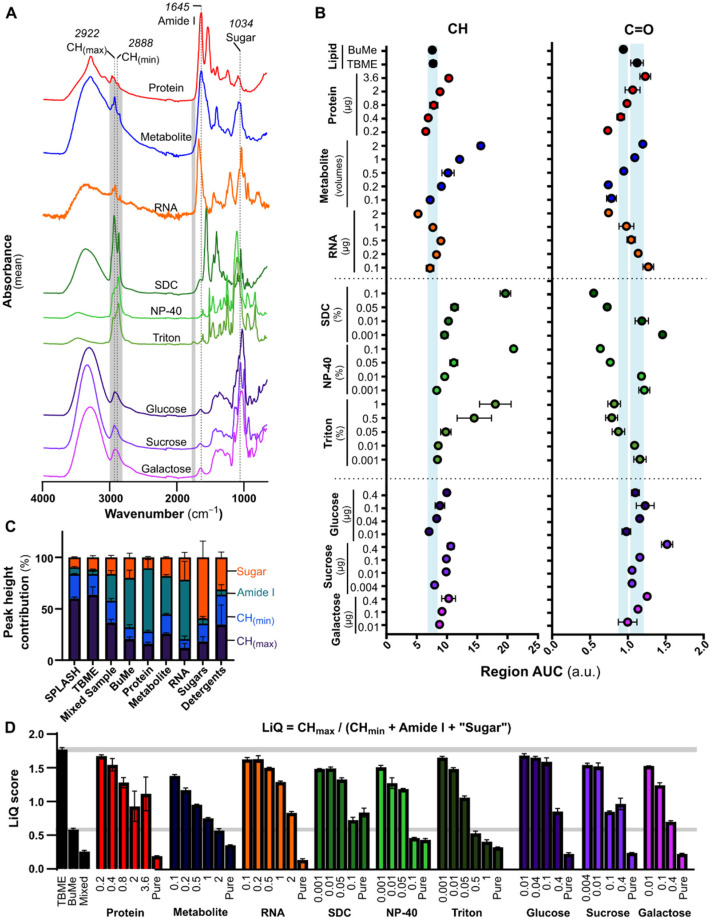
Lipid Quality (LiQ) score development. (**A**) FTIR spectra of common lipid contaminants: protein (1 µg/µL) extracted from cultured cells, RNA (1 µg/µL) from a synthesized primer, metabolites extracted from cultured cells, the detergents Triton X-100, NP-40, and sodium deoxycholate (SDC, all 1% *v*/*v*), and the sugars sucrose, glucose, and galactose (all 1 µg/µL). Lipid characteristic regions are highlighted in gray, and characteristic contaminant bands are labeled. Three technical measurements were acquired and averaged. (**B**) Quantification of lipid peaks (CH and C=O) by AUC measurements revealed the effects of contaminants on accurate quantitation. Varying concentrations of contaminant were spiked into pure lipid samples (1 µg per measurement, from TBME extraction). Three technical replicates were acquired for each sample, and error bars represent standard deviation. Light blue regions highlight the expected AUC from TBME- and BuMe-extracted lipids. (**C**) Identification and contribution of prominent peaks in FTIR spectra of biological materials. Prominent spectral regions identified in panel A were evaluated by peak heights and averaged across all replicates. All contaminants considered, including glucose, sucrose, and galactose sugars, and the detergents SDC, Triton-X100, and NP-40, were averaged across the group. The contribution of each peak height is summarized as an averaged percentage and standard deviation. Each contaminant without lipid was also measured (“neat”). (**D**) Contaminated lipid samples evaluated using the LiQ score. Peak heights at CH_max_ were compared to the sum of the CH_min_, Amide I and sugar peaks. Gray highlighted regions represent the expected LiQ scores for pure lipid or BuMe extracted lipids, within one standard deviation.

Spectroscopic methods are employed in DNA/RNA sequencing and proteomics workflows for quality and/or quantity measures prior to omics analysis. Drawing inspiration from NanoDrop technology, which evaluates RNA or DNA-specific signatures compared to contaminating molecules in a ratiometric measure (e.g., A260/280 ratio), we sought to delineate spectral features that can be used as a measure of contamination and lipid quality. Firstly, we catalogued the FTIR spectra of common contaminants that may be present in lipid extracts, either as a consequence of a sampling error allowing for the coextraction of biological metabolites and protein, or through technical or procedural additives used in upstream processes. Namely, detergents used in sample lysis and storage, or plasticware preparation and sucrose for specific structure separation (e.g., organelles) can be coextracted due to their lipid-like properties or overwhelming abundance, respectively. Additionally, metabolites and proteins may be present in prepared lipid samples during lipidomics preparation due to user error. Hereby, we evaluated common lysis detergents Triton X-100, NP-40, and sodium deoxycholate (SDC) (all 1% *v*/*v*), the sugars sucrose, glucose, and galactose (all 1 µg/µL), and possible coextracted biological compounds including protein (1 µg/µL) from cultured cells, RNA (1 µg/µL) from a synthesized primer and a metabolite fraction extracted from cultured cells (containing undetermined saccharide and nucleic acid). The lipid signature CH and C=O regions are shaded in grey in Figure 3A for comparison. Like lipids, some detergents display large CH peaks; in lipids, these peaks are much more sharply defined. This is most consistently characterized by a larger hydrocarbon minimum (“CH_min_”, 2888 cm^−1^, Figure 3A). Both protein and nucleic acid samples showed distinctive peaks in the 1700–1550 cm^−1^ region (labeled “Amide I”, Figure 3A), typically considered to be a characteristic of amide and amine functional groups [15]. The most distinctive feature in sugar-containing samples is the large CO-stretching contribution (a mix of COH and single bonded CO, labeled “sugar” region, 1200–1000 cm^−1^). Metabolite samples presented with features of pure sugar and RNA spectra. 

Notably, all tested compounds show hydrocarbon-specific peaks (3000–2800 cm^−1^), a broad OH/NH peak (3500–2500 cm^−1^), or a large amine/amide peak (1645 cm^−1^ maxima, variable range), all of which could contribute background signal to the lipid-specific CH and C=O peaks. To estimate the effect that these contaminants have on the lipid quantitation, we conducted a titration assay by combining varying concentrations of these contaminants in a constant amount of lipid (1 µg/µL). AUCs were calculated for both the CH and C=O regions without baseline subtraction (Figure 3B). Large amounts of contaminating material led to inaccurate AUCs in both regions, highlighting the need for sample contamination/purity assessment prior to quantification.

To facilitate high-throughput lipid quality assessment, we developed a sample Lipid Quality (LiQ) score algorithm based on peak heights, which can be applied automatically to exported spectra in Excel. This score evaluates the ratio between lipid peaks and non-lipid spectral features present in common contaminants. We considered the common spectral features for biological molecules (Table 2), and selected the following peak changes identified in the contaminant dataset: CH-stretching peak maxima (CH_max_, 2922 cm^−1^), CH minima (CH_min_, 2888 cm^−1^), amide I maxima (1645 cm^−1^), and “sugar” maxima (1034 cm^−1^, COH and CO vibration). As shown in Figure 3C, the pure lipid samples SPLASH and TBME are more enriched in CH regions than other sample types, except for detergents (Figure 3C). Compared to lipids, detergents have a higher proportion of CH_min_ than CH_max_. Therefore, we devised the LiQ Score as the peak height of the CH_max_ divided by (CH_min_ + amide I maxima + “sugar” maxima). While species of biological lipids will also contain amines, sugars and phosphodiester functional groups, the relative abundance of these groups does not outweigh or exceed the contribution of hydrocarbon or lipid ester bond due to the chemical stoichiometry of these molecules. Thus, an accepted range that evaluates the tipping point between lipid sample and non-lipid content was determined. The LiQ Score was calculated for the range of samples evaluated so far (Figure 3D). TBME extracted lipids generated a LiQ Score of 1.7 ± 0.03, which does not overlap with the contaminated samples even at the lowest contaminant concentrations. BuMe extraction produced a lower LiQ Score of 0.5 ± 0.01, reflecting metabolite coextraction. For analysis of suspect pure lipids, a LiQ score over 1.7 is recommended as an index for high-quality lipid preparation. Due to varying metabolite quantities in different samples types [16], monophasic extractions are likely to have differing LiQ scores, where a LiQ of 0.35 corresponds to metabolite-only samples. High-concentration galactose (0.4 µg galactose/µg lipid) and high-concentration Triton-X100 (0.5% Triton with 1 µg lipid) produced similar LiQ scores to the BuMe lipids; however, more detailed inspection of the spectra is needed to delineate the specific nature of the contamination. We provide some methods for achieving this in Figure S1.

### 3.3. Evaluation of Lipid Quality and Quantity in Human Plasma Samples

**Figure 4:**
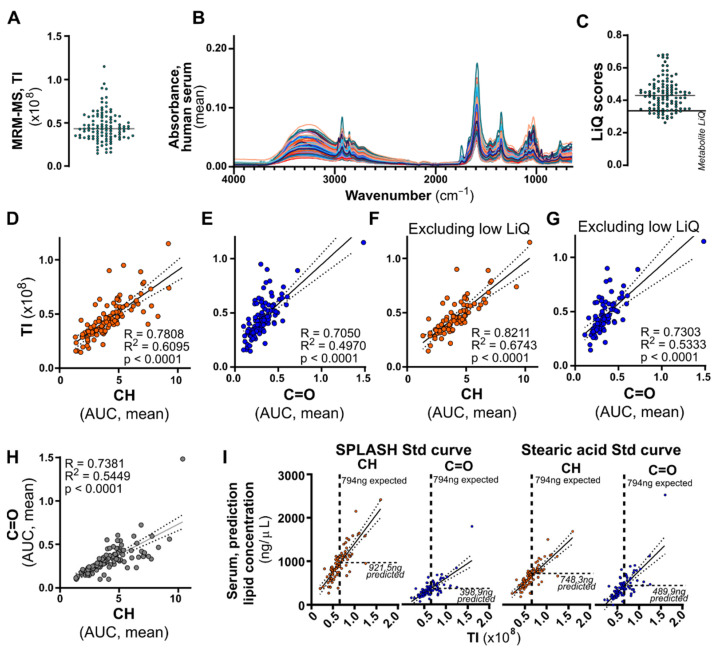
Comparison of ATR-FTIR quantification and mass spectrometry, and quality control analysis of human plasma lipidomics samples. Lipids were extracted from 107 human plasma samples using the BuMe method and analyzed using ATR-FTIR spectroscopy and multiple-reaction monitoring mass spectrometry (MRM-MS). (**A**) Jitter plot of all total intensities (TI) measured by mass spectrometry for each sample. (**B**) Absorbance spectra acquired by ATR-FTIR spectroscopy for 107 human plasma lipidomics samples. Technical replicates were averaged. (**C**) Lipid quality (LiQ) score for each plasma sample. The LiQ score for pure metabolite samples (LiQ = 0.351) is shown as a thick black line. (**D**–**G**) The CH and C=O regions were measured by AUC and compared to MS by simple linear regression (R^2^ and *p* values) and Pearson correlation (R values). (**D**,**E**) Comparison for all 107 human plasma lipidomics samples. (**F**,**G**) Comparison for 82 human plasma lipid quantification after removal of low-LiQ-scoring samples. (**H**) Linear relationship between the AUC of the CH and C=O regions detected by FTIR spectrometry of human plasma lipid samples. (**I**) Absolute quantification of human plasma lipid samples using SPLASH Lipidomics or stearic acid standard curves. The CH (orange) and C=O (blue) regions were measured for both standards and compared to TI by linear regression analysis. Quantification of a 794 ng/µL lipid control was also carried out via MS and compared to the FTIR regression equation to calculate predicted absolute quantities by different standards and regions.

The methods we developed to assess lipid quality and quantity were evaluated on a batch of 107 human plasma lipid samples. They were analyzed using a high-throughput lipidomics method that measures all major plasma lipid classes via multiple reaction monitoring-MS after BuMe extraction [10], with equal plasma volume loading. The total intensity (TI) from MRM-MS data shows a spread of values, indicative of differing plasma lipid concentrations and the lack of equal lipid loading (Figure 4A). After MS data acquisition, ATR-FTIR spectra were acquired for each sample, averaged, and shown in Figure 4B. For each sample, technical replicates were averaged and then the LiQ score and lipid quantity were calculated using CH, C=O, and both stearic acid and SPLASH LipidoMix calibration curves, with and without exclusion of low-scoring samples. The mean LiQ score for all samples was 0.4353 (range 0.2631–0.6804 (Figure 4C). As a conservative approach for excluding low purity samples, we used the pure metabolite LiQ score of 0.3514 as a cut-off (thick line in Figure 4C), which flagged 25 samples as low quality. Comparing quantification by CH or C=O region with MS total intensities identified linear relationships for each method, with a slightly stronger trend in CH regions (R = 0.7808, R^2^ = 0.6095, *p* < 0.0001) than C=O (R = 0.705, R^2^ = 0.4970, *p* < 0.0001), (Figure 4D,E). Excluding low-quality samples led to improvements in the linear relationship seen for both the CH (R = 0.8211, R^2^ = 0.6743, *p* < 0.0001) and C=O (R = 0.7303, R^2^ = 0.5333, *p* < 0.0001) regions (Figure 4F,G).

The total lipid quantity for each sample was calculated using both stearic acid and SPLASH LipidoMix calibration curves, using either the CH or C=O regions (Figure 4H). The two calibration curves generated similar results, but the calculated lipid quantity is highly dependent on the spectral region used, with the C=O region consistently predicting a lower quantity than the CH region. To determine whether CH or C=O is more accurate for absolute quantification, we ran 794 ng of SPLASH LipidoMix on MS, then used either the SPLASH LipidoMix or stearic calibration curve to calculate lipid amount. As shown in Figure 4I, the CH AUC provides a closer estimate (921.5 ng and 748.3 ng) compared to the C=O region (398.9 ng and 489.9 ng). While SPLASH LipidoMix calibration overestimated the quantity by 16%, stearic acid underestimated the quantity by 9%. Taken together, the CH region without baseline correction provides the most accurate lipid quantification in high quality samples without interfering contaminants.

## 4. Discussion

Here, we present a comprehensive evaluation of ATR-FTIR for complex lipid extract quantification and propose an integrated, simple, sample-conserving method to assist in lipidomics sample quality assessment prior to mass spectrometry analysis. There are several important reasons to evaluate lipid sample quality early in the lipidomics workflow. Firstly, certain contaminants, such as detergents, can confound MS data and even impact LC-MS performance for later samples in the sequence. Early detection of contaminated samples will allow elimination of these sample from the analysis, or re-extraction of the sample. Secondly, contamination can also confound total lipid quantification by FTIR, as illustrated in Figure 3 and applied in Figure 4. While we have devised a simple LiQ Score to facilitate screening for exclusion of low-quality samples, closer analysis and interpretation of spectral features can provide additional information on the potential contaminants in these samples. To this end, we have offered three follow-on ratiometric analyses in Figure S1. These additional peak height ratios compare the contribution of lipid peak versus detergent, protein, or sugar peaks individually to clarify the composition of the sample and potentially inform the source of contaminants. 

Our evaluation of lipid quantification by FTIR highlights a detrimental impact of baseline subtraction on the CH region AUC, which we determined to be more accurate than the alternative C=O peak. Baseline subtraction is also referred to as baseline correction, or anchoring, and can be a default parameter in FTIR spectral processing software [7,14]. While useful in some applications, quantification of peaks in complex mixtures with overlapping spectra faces augmentation of the local baseline absorbance. This bisects the target peak and results in partial exclusion of peak areas that are masked by the non-target peaks, leading to underestimates in terms of quantity. More sophisticated methods such as spectral deconvolution could be employed to accurately separate the target from non-target peaks, as demonstrated in inorganic chemistry applications [25]. However, attempts for complex biological features have been met with difficulty [21]. We caution users about the use of local baseline correction for FTIR AUC-based lipid quantification calculations, especially in instances of non-pure lipid samples. 

Our detailed assessment of the calibration curve of a simple lipid (stearic acid) and a complex (SPLASH LipidoMix) lipid mixture showed a similar signal-to-noise ratio, linearity, limit of detection, and quantification. SPLASH LipidoMix is produced to reflect the human plasma composition, comprising of physiologically accurate concentrations of phospholipids, sphingolipids, cholesterol, cholesterol esters, and glycerides, whereas stearic acid merely reflects a simple fatty acid of 18-carbon length. The difference in lipid composition of the two standards is evident in the full spectra (Figure 2A), where amine, phosphodiester, and sugar groups are present in the complex mixture reflecting an expected biological sample. Prediction of lipid quantities was not impacted by this added complexity. Instead, it was highly dependent on the hydrocarbon chain length and number of chains (bound to head group by the lipid ester group) of the chosen lipid standards and sample. Use of a simple lipid standard should be chosen to reflect the average lipid chain and number of the sample for the closest accuracy due to this relationship. Other FTIR-based analyses have been proposed to delineate lipid class composition and shifts in lipid spectra in disease [19,26], whereby use of different species of lipid as standard may also provide some frame of reference for specific lipid classes or disease information. 

Compared to the commercial product DirectDetect, which uses transmission mode for spectra acquisition, there are several advantages of our proposed ATR-FTIR lipid quality and quantity assessment workflow. DirectDetect outputs the calculated lipid quantity using the AUC from the CH region 2870–2840 cm^−1^ with baseline anchoring, which is narrower than our CH band (3000–2800 cm^−1^), and the recommendation to not baseline subtract. As far as we are aware, DirectDetect does not allow the opportunity to modify the quantification method or ranges to customize use [27]. The stated limit of detection for DirectDetect is 250 ng/μL, which is ten-fold higher than the proposed ATR-FTIR workflow [7]. No additional consumables are required in our workflow, although analysis is carried out one sample at a time. The DirectDetect sample cards hold four samples at a time, but it takes several minutes to read each card [27]. Our workflow requires approximately 2 min for each sample. While 1 µL of lipid sample was demonstrated as feasible in the ATR-FTIR workflow as an effort to minimize sample wastage, pipetting of small volumes of organic solvent can be difficult. However, analysis of the ATR-FTIR technical replicates from the 107 plasma samples showed coefficient of variance of <5% (Figure S2), indicative of high reproducibility. For the best accuracy, we recommend using air-displacement pipettes, keeping samples at a low temperature during workflow, and conditioning the pipette tip by aspirating and dispensing the sample at least twice before transferring sample to the detector. Technical replicates may also be advised to gauge pipetting accuracy. 

One of the benefits of the ATR-FTIR workflow is the customization of the parameters for other applications. Overall, this analysis relies on generalization of lipid structure, using only the hydrocarbon CH and ester C=O regions as proxy for lipid content, which allows this method to be used broadly. This is similar to UV/VIS spectroscopy for RNA analysis using only the A260 measure for nucleic acid despite variation in spectral peaks for each nucleotide [5]. Here, we assessed the CH and C=O region as two possible generalized ranges for lipid quantification based on their prevalence in complex biological samples for lipidomics experiments. However, some applications and sample sources may require adjustment of these parameters. While lipidomics samples for mass spectrometry are prepared in organic solvent, other applications may find lipid in aqueous suspensions and thus may be more suitable for using the C=O region for quantification due to the overlapping water signal hindering CH region detection [28]. Unsaturation of lipids results in an additional CH peak between 3010–3000 cm^−1^ and a reduction in the CH-region (3000–2800 cm^−1^, Figure S3). While in human-derived samples, the unsaturation signal is minor compared to saturated signals, samples rich in unsaturated lipid may require an extension of the CH region to encompass this 3010 cm^-1^ peak. This analysis of CH peaks may be extended as a method for measuring lipid unsaturation in the future. Additionally, fatty acids contain a carboxyl peak (~1710 cm^−1^) as opposed to a lipid ester peak (~1740 cm^−1^), and therefore extension of the C=O region may be beneficial for highly fatty-acid-enriched samples. 

## 5. Conclusions

This study establishes the key technical parameters for use of ATR-FTIR as a simple method for complex lipid sample quality control and quantification, highlighting contaminants and local baseline correction as sources of error in lipid quantification. Furthermore, we developed the simple LiQ score to facilitate sample quality screening, and also offer more complex ratiometric assessments to further characterize contaminants. As this validated method fills the lipid sample quality control gap, requires no additional consumables, and uses minimal sample, we anticipate it will be useful in lipidomics workflows.

## Figures and Tables

**Table 1:** Characteristics of two different ATR-FTIR lipid calibration curves.

	Stearic Acid		SPLASH LipidoMix	
	CH	C=O	CH	C=O
**SNR**	2.5	4.4	3.3	3.1
**LOD**	12.7 ng	18.8 ng	11.6 ng	29.5 ng
**LOQ**	42.5 ng	62.8 ng	38.7 ng	98.6 ng
**Linear equation**	0.005162x + 0.2286	0.000464x + 0.1159	0.004206x + 0.215	0.000681x + 0.05761
**Standard error of slope**	6.24 × 10^−5^	3.41 × 10^−5^	8.12 × 10^−5^	8.68 × 10^−6^

CH (3000–2800 cm^−1^), C=O (1760–1710 cm^−1^). SNR—signal-to-noise ratio. LOD—limit of detection, calculated by 3 × Dblank /linear regression gradient. LOQ—limit of quantitation, calculated by 10 × SDblank/gradient.

**Table 2:** Distinguishing spectral features in biological molecules. A collection of the most distinctive vibrational frequencies (in wavenumber) detected in tested biological molecules, with biological interpretations and chemical structure descriptions.

Structural and Compositional Regions	Wavenumber (cm^−1^)	Molecule Type	References
**Hydrocarbon**			[17,18]
**CH3** (stretching, asymmetric)	2958	Enriched in lipid, detergent. Contained in most organic molecules. Alkenes increase in unsaturated lipid.	
**CH2 (“CH_max_”**, stretching, asymmetric)	2922		
**CH3 (“CH_min_”**, stretching, symmetric)	2888		
**CH** (alkane, stretching, asymmetric)	2925		
**CH** (alkene, stretching)	3020		
**CH** (bending)	1450		
**Carbonyl** (C=O)			[19,20]
**Carboxyl** (C=OOH)	1710	Fatty acids (carboxyl) Lipids (ester)	
**Ester** (C=OOC)	1740		
**Amide**			[21,22]
**Amide A** (NH-stretching)	3525	Protein, peptide (amide), nucleic acids (amine)	
**Amide I** (**“amide I maxima”**, NH-bending)	1645		
**Amide II** (NH-bending)	1550		
**Fingerprint region**			[22,23,24]
**COH** (hydroxyl, stretching)	1160	Saccharide, glycosphingolipids, nucleic acids, phospho-groups.	
**CO** (**“sugar maxima”**, stretching)	1034, 1160		
**PO2-** (stretching, asymmetric)	1245		
**PO2-** (stretching, symmetric)	1080		

## Data Availability

FTIR spectra are available via Zenodo (DOI:10.5281/zenodo.6592243). Mass spectrometry data are available via Panorama at https://panoramaweb.org/LiQscoreEvaluation.url, accessed on 1 September 2022.

## Supplementary Materials

The following supporting information can be downloaded at: https://www.mdpi.com/article/10.3390/biom12091265/s1, Figure S1: Complementary measurements for LiQ score quality control analysis; Figure S2: Variation in technical and biological replicates. Figure S3: Variations in the CH region by unsaturated fatty acids.

## References

[1] Finkelstein J., Heemels M.T., Shadanm S., Weiss U. (2014). Lipids in health and disease. Nature.

[2] Fahy E., Subramaniam S., Brown H.A., Glass C.K., Merrill A.H., Murphy R.C., Raetz C.R., Russell D.W., Seyama Y., Shaw W. (2005). A comprehensive classification system for lipids. J. Lipid Res..

[3] Molendijk J., Robinson H., Djuric Z., Hill M.M. (2020). Lipid mechanisms in hallmarks of cancer. Mol. Omics.

[4] Kofeler H.C., Ahrends R., Baker E.S., Ekroos K., Han X., Hoffmann N., Holčapek M., Wenk M.R., Liebisch G. (2021). Recommendations for good practice in MS-based lipidomics. J. Lipid Res..

[5] Fischer J. (1995). Specific detection of nucleotides, creatine phosphate, and their derivatives from tissue samples in a simple, isocratic, recycling, low-volume system. LC-GC Int..

[6] Desjardins P., Conklin D. (2010). NanoDrop microvolume quantitation of nucleic acids. J. Vis. Exp..

[7] Strug I., Utzat C., Cappione A., Gutierrez S., Amara R., Lento J., Capito F., Skudas R., Chernokalskaya E., Nadler T. (2014). Development of a Univariate Membrane-Based Mid-Infrared Method for Protein Quantitation and Total Lipid Content Analysis of Biological Samples. J. Anal. Methods Chem..

[8] Merck Millipore (2013). Simplified Analysis of Lipid or Detergent Content in Biological Samples Using the IR-Based Direct Detect^®^ Spectrometer.

[9] Tanneru S., Steele P. (2014). Pretreating bio-oil to increase yield and reduce char during hydrodeoxygenation to produce hydrocarbons. Fuel.

[10] Huynh K., Barlow C.K., Jayawardana K.S., Weir J.M., Mellett N.A., Cinel M., Magliano D.J., Shaw J.E., Drew B.G., Meikle P.J. (2019). High-Throughput Plasma Lipidomics: Detailed Mapping of the Associations with Cardiometabolic Risk Factors. Cell Chem. Biol..

[11] Peng B., Ahrends R. (2016). Adaptation of Skyline for Targeted Lipidomics. J. Proteome Res..

[12] R Core Team (2017). R: A Language and Environment for Statistical Computing.

[13] Shrivastava A., Gupta V.B. (2011). Methods for the Determination of Limit of Detection and Limit of Quantitation of the Analytical Methods. Chron. Young Sci..

[14] Provis-Evans C.B., Farrar E.H., Grayson M.N., Webster R.L., Hill A.K. (2020). Highly Sensitive Real-Time Isotopic Quantification of Water by ATR-FTIR. Anal. Chem..

[15] Arunkumar R., Drummond C.J., Greaves T.L. (2019). FTIR Spectroscopic Study of the Secondary Structure of Globular Proteins in Aqueous Protic Ionic Liquids. Front. Chem..

[16] Martins Conde P., Pfau T., Pires Pacheco M., Sauter T. (2021). A dynamic multi-tissue model to study human metabolism. NPJ Syst. Biol. Appl..

[17] Versteegh G., Houben A.J.P., Zonneveld K. (2020). Better molecular preservation of organic matter in an oxic than in a sulfidic depositional environment: Evidence from Thalassiphora pelagica (Dinoflagellata, Eocene) cysts. Biogeosciences.

[18] Talukdar J., Kalita M.C., Goswami B.C., Hong D.D., Das H.C. (2014). Liquid Hydrocarbon Production Potential of a Novel Strain of the Microalga Botryococcus braunii: Assessing the Reliability of in Situ Hydrocarbon Recovery by Wet Process Solvent Extraction. Energy Fuels.

[19] Forfang K., Zimmermann B., Kosa G., Kohler A., Shapaval V. (2017). FTIR Spectroscopy for Evaluation and Monitoring of Lipid Extraction Efficiency for Oleaginous Fungi. PLoS ONE.

[20] Filopoulou A., Vlachou S., Boyatzis S.C. (2021). Fatty Acids and Their Metal Salts: A Review of Their Infrared Spectra in Light of Their Presence in Cultural Heritage. Molecules.

[21] Fellows A.P., Casford M.T.L., Davies P.B. (2020). Spectral Analysis and Deconvolution of the Amide I Band of Proteins Presenting with High-Frequency Noise and Baseline Shifts. Appl. Spectrosc..

[22] Rolim T., Cancino J., Zucolotto V. (2015). A nanostructured genosensor for the early diagnosis of systemic arterial hypertension. Biomed. Microdevices.

[23] Ensari Ö. (2012). Polyethylene Glycol-Sugar Composites as Shape Stabilized Phase Change Materials for Thermal Energy Storage. Polym. Compos..

[24] Wang T.D., Triadafilopoulos G., Crawford J.M., Dixon L.R., Bhandari T., Sahbaie P., Friedland S., Soetikno R., Contag C.H. (2007). Detection of endogenous biomolecules in Barrett’s esophagus by Fourier transform infrared spectroscopy. Proc. Natl. Acad. Sci. USA.

[25] Brangule A., Gross K.A. (2015). Importance of FTIR Spectra Deconvolution for the Analysis of Amorphous Calcium Phosphates. IOP Conf. Ser. Mater. Sci. Eng..

[26] Depciuch J., Zawlik I., Skrzypa M., Pająk J., Potocka N., Łach K., Bartosik-Psujek H., Koziorowska A., Kaznowska E., Cebulski J. (2019). FTIR Spectroscopy of Cerebrospinal Fluid Reveals Variations in the Lipid: Protein Ratio at Different Stages of Alzheimer’s Disease. J. Alzheimers Dis..

[27] Merck Millipore (2014). Direct Detect Spectrometer User Guide.

[28] Tranter G.E., Lindon J.C., Tranter G.E., Koppenaal D.W. (2017). FTIR Spectroscopy of Aqueous Solutions. Encyclopedia of Spectroscopy and Spectrometry.

