# Supplementary material for: Rapid Assessment of Lipidomics Sample Purity and Quantity Using Fourier-Transform Infrared Spectroscopy
Source: Biomolecules. 2022 Sep 8;12(9):1265. doi: 10.3390/biom12091265 (PMC9496531; doi:10.3390/biom12091265)
Supplement: Supplementary file 1 [file biomolecules-12-01265-s001.zip › biomolecules-1851132-supplementary.pdf]

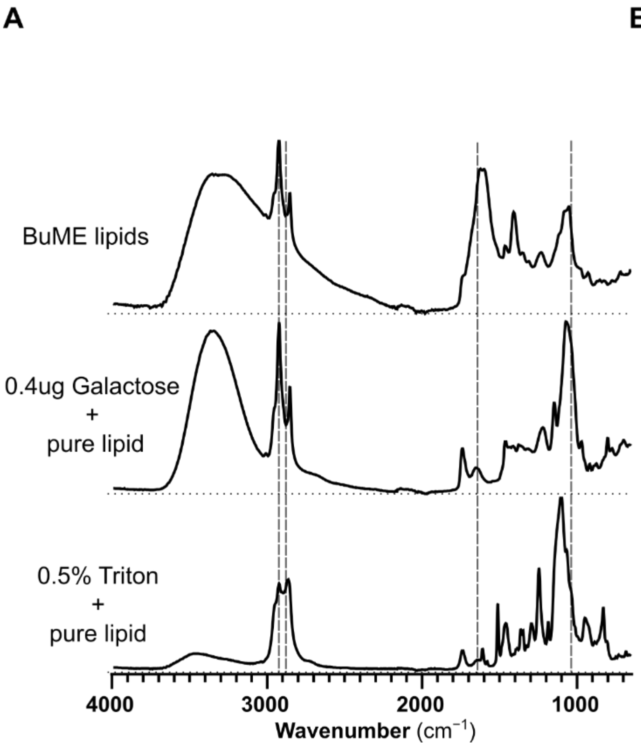

B

LiQ score	Spectral feature ratio		
$\frac{CH_{max}}{(CH_{min} + \text{Amide} + \text{"Sugar"})}$	$\frac{CH_{max}}{CHmin}$	$\frac{CH_{max}}{\text{Amide}}$	$\frac{CH_{max}}{\text{"Sugar"}}$
0.58 (+/− 0.02)	1.76 (+/− 0.03)	1.67 (+/− 0.06)	2.40 (+/− 0.10)
0.69 (+/− 0.01)	2.28 (+/− 0.03)	7.50 (+/− 0.61)	1.19 (+/− 0.05)
0.53 (+/− 0.02)	1.09 (+/− 0.09)	10.96 (+/− 0.54)	1.18 (+/− 0.09)

Figure S1: Complementary measurements for LiQ score quality control analysis. A) Stacked spectra of BuMe extracted lipids, and lipid samples contaminated with 0.5% Triton-X100, or 0.4µg galactose per 1µg TBME extracted lipid. Vertical dotted lines highlight the important spectral regions, specifically, CH_{max}, CH_{min}, Amide I, and "Sugar" regions. **B)** Comparison of LiQ scores and spectral feature ratios as metrics of lipid purity. Despite similar LiQ scores across each spectrum, the ratios of spectral features indicate the presence of contaminants, which is not ideal.

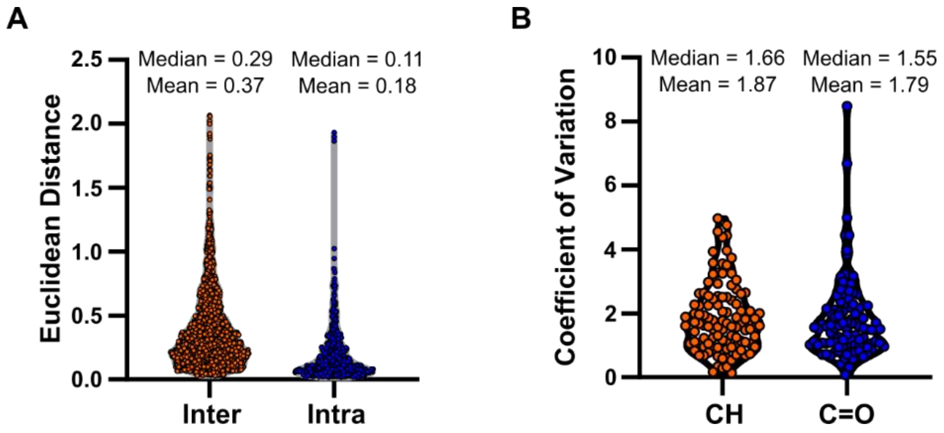

Figure S2: Variation in technical and biological replicates. 3–5 technical replicates were measured for all 107 human plasma samples. **A)** The variation between technical replicates (intra-sample variation) and between biological replicates (inter-sample variation) measured by Euclidean distance. **B)** Coefficient of variation for the measured AUCs for CH and C=O region for each biological replicate.

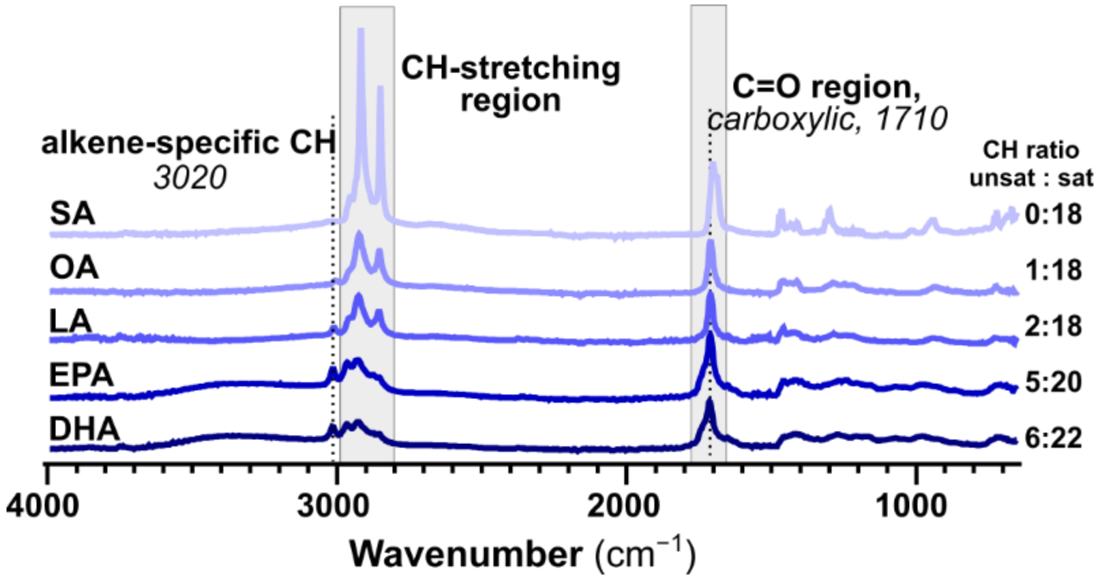

Figure S3: Variations in the CH region by unsaturated fatty acids. FTIR spectra of polyunsaturated fatty acids. Unsaturation to chain ratio shown on the right. Equal amount of lipid was measured for each (625ng). Fatty acids measured are Stearic acid (SA), Oleic acid (OA), Linoleic acid (LA), Eicosapentaenoic acid (EPA), Docosahexaenoic acid (DHA), all purchased from Sigma-Aldrich. Unsaturated lipids gain an alkene specific CH peak while reducing the prevalent peaks in the saturated lipid CH region, while lipid carboxyl regions (C=O) remain uniform.
